# Laparoscopic Cholecystectomy Mirizzi Syndrome Due to a Long Dilated Cystic Duct Stump as a Result of an Impacted Stone: A Diagnostic and Surgical Pitfall

**DOI:** 10.7759/cureus.91956

**Published:** 2025-09-10

**Authors:** Hassan M Lameen, Adham Darweesh, Abdulqadir J Nashwan, Usamah Al-Anbagi

**Affiliations:** 1 Clinical Imaging Department, Hamad Medical Corporation, Doha, QAT; 2 Nursing and Midwifery Research Department, Hamad Medical Corporation, Doha, QAT; 3 Internal Medicine Department, Hazm Mebaireek General Hospital/Hamad Medical Corporation, Doha, QAT

**Keywords:** cystic duct stump, endoscopic retrograde cholangiopancreatography (ercp), magnetic resonance cholangiopancreatography (mrcp), mirizzi syndrome (ms), post-cholecystectomy

## Abstract

Mirizzi syndrome (MS) is an uncommon complication of gallstone disease in which an impacted stone in the cystic duct or gallbladder neck leads to external compression of the common bile duct (CBD). Its clinical and imaging features often mimic other hepatobiliary conditions, making preoperative diagnosis challenging and increasing the risk of biliary injury. We report the case of a 34-year-old male who presented with sudden-onset right upper quadrant pain. His history of gallstones and examination findings suggested acute calculous cholecystitis. Laboratory tests revealed elevated liver enzymes, while abdominal ultrasound demonstrated gallstones without CBD dilation. He underwent a laparoscopic cholecystectomy on hospital day three. Intraoperative findings included a distended gallbladder with a dilated cystic duct and CBD. Postoperative magnetic resonance cholangiopancreatography (MRCP) revealed a long cystic duct stump with an impacted stone causing compression of the CBD and proximal biliary dilation, consistent with MS. Subsequent endoscopic retrograde cholangiopancreatography (ERCP) confirmed the diagnosis, and sphincterotomy with CBD stenting was performed. The patient recovered uneventfully and remained stable on follow-up. This case underscores the importance of maintaining a high index of suspicion for MS, particularly in patients with gallstones presenting with atypical features or persistent biliary dilation. Early identification and multidisciplinary management are crucial for preventing complications and ensuring optimal outcomes.

## Introduction

Mirizzi syndrome (MS) is a rare complication of gallstone disease caused by an impacted stone in the cystic duct or gallbladder infundibulum that leads to external compression of the common hepatic duct (CHD) or common bile duct (CBD) [1]. This obstruction often results in chronic inflammation and can progress to cholecystobiliary fistulas in advanced cases [1]. MS occurs in approximately 0.05%-4% of patients undergoing cholecystectomy, with a higher prevalence in females, paralleling the overall incidence of gallstones [1]. Clinically, MS can mimic acute cholecystitis, choledocholithiasis, or even biliary malignancy, making preoperative diagnosis challenging and increasing the risk of bile duct injury during surgery.

Despite advances in imaging modalities, such as magnetic resonance cholangiopancreatography (MRCP) and endoscopic retrograde cholangiopancreatography (ERCP), many cases remain undiagnosed until surgery. Maintaining a high index of suspicion is crucial, especially in patients with atypical presentations or persistent biliary symptoms. This report presents the case of a young male whose initial diagnosis of acute calculous cholecystitis was revised to MS after intraoperative and advanced imaging findings. Highlighting this case emphasizes the importance of thorough perioperative evaluation and a multidisciplinary approach to optimize patient outcomes.

## Case presentation

History

A 34-year-old male presented with a chief complaint of sudden-onset right upper quadrant pain of one day’s duration. The pain was continuous, sharp, and not related to food intake. It was not relieved by changes in position nor did over-the-counter analgesics provide any relief. The patient also reported anorexia but denied heartburn, vomiting, or changes in bowel habits. There was no associated fever or chills, and no signs of systemic infection.

The patient had a known history of gallstones and reported multiple prior episodes of similar, but less severe, pain over the past year, which resolved spontaneously without medical intervention. He denied any history of jaundice, pruritus, or other related symptoms. His past medical history was otherwise unremarkable, with no chronic illnesses. He had no history of previous surgeries and no known drug allergies.

Examination

Upon examination, the patient was afebrile (36.6°C) with stable vital signs: heart rate of 52 beats/min, respiratory rate of 17 breaths/min, blood pressure of 131/63 mmHg, and oxygen saturation of 100% on room air. Abdominal examination revealed tenderness in the right upper quadrant with a positive Murphy’s sign. The abdomen was flat, without scars or distension. There was no rigidity, no palpable masses, and no hepatomegaly. Percussion was negative for ascites, bowel sounds were normal, and no vascular bruits were detected. The remainder of the systemic examination was unremarkable.

Management and follow-up

Initial laboratory investigations showed elevated liver enzymes, normal inflammatory markers, normal renal function, and normal bilirubin levels (Table 1).

**Table 1:** Laboratory parameters on admission and at discharge CRP: C-reactive protein; ALT: Alanine aminotransferase; AST: Aspartate aminotransferase; PT: Prothrombin time; INR: International normalized ratio; APTT: Activated partial thromboplastin time

Parameters	On admission	On discharge	Reference values
Total leukocytes	4.7	5.4	(6.2 x 10^3^/uL)
Hematocrit	41.7	42.3	(40-50%)
Hemoglobin (gm/dL)	14.7	14.4	(13-17 gm/dL)
Platelet (x 10^3^/uL)	215	318	(150-410 x 10^3^/uL)
CRP (mg/L)	3.1	<2	(0-5 mg/L)
Serum urea (mmol/L)	6.2	5.9	(2.5-7.8)
Serum creatinine (umol/L)	90	85	(62-106)
Serum potassium K (mmol/L)	3.9	4.1	(3.5-5.3)
Serum sodium (mmol/L)	136	136	(133-146)
Serum total protein (gm/L)	69	72	(60-80)
Serum albumin (gm/L)	34	33	(35-50)
ALT (IU/L)	522	289	(0-41)
AST (IU/L)	335	160	(0-41)
Alkaline phosphatase (U/L)	136	243	(40–129)
Serum total bilirubin (mg/dL)	16	21	(0-21)
PT (seconds)	-	13.5	(9.4-12.5 seconds)
INR	-	1.2	<1
APTT (seconds)	-	30.9	(25.1-36.5 seconds)

**Figure 1:**
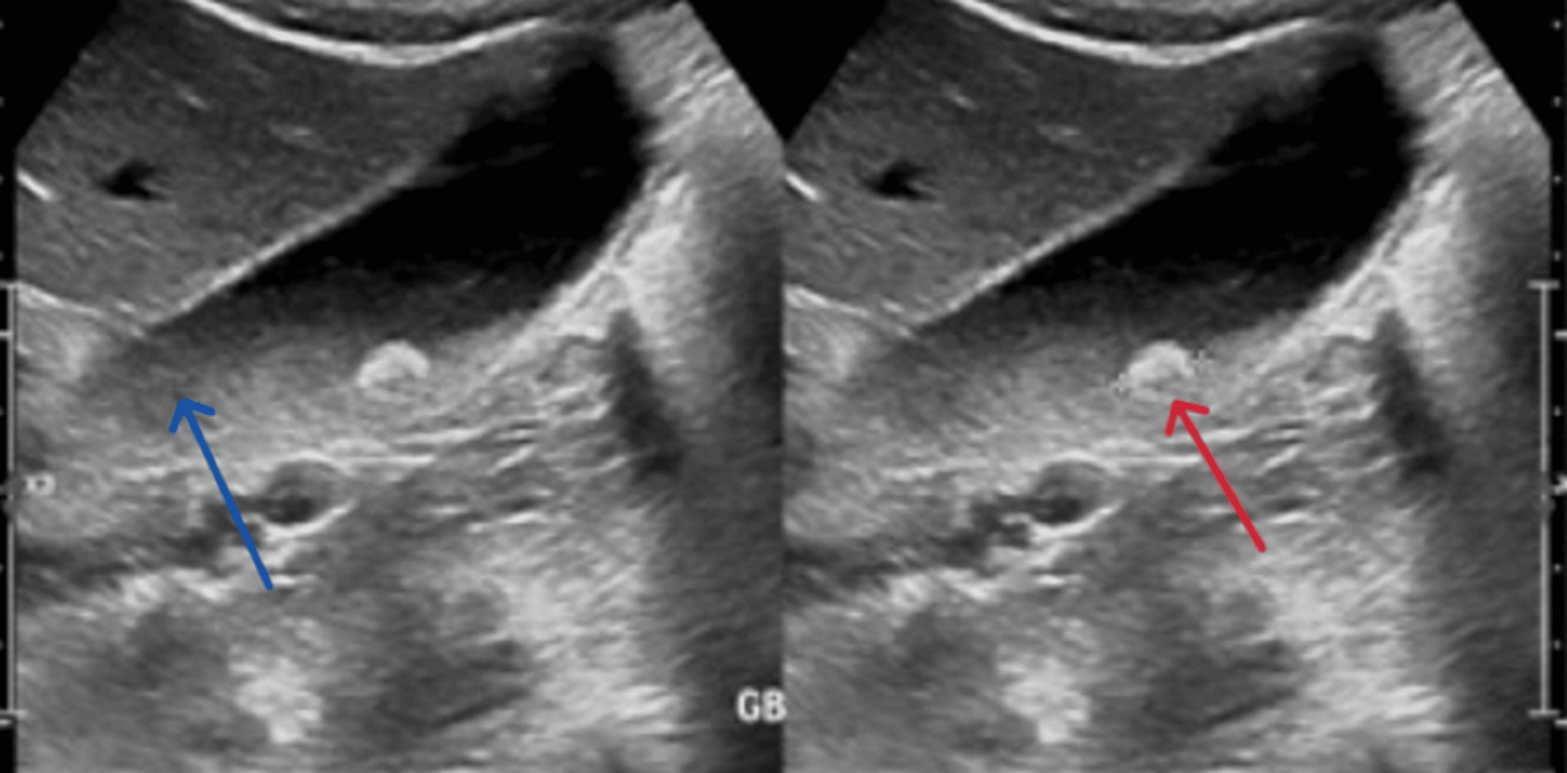
Transabdominal ultrasound findings of the gallbladder Transabdominal ultrasound demonstrates a distended gallbladder with an 8 mm gallstone (red arrow) and associated thick sludge formation (blue arrow). Mild gallbladder wall thickening is also noted, consistent with acute calculous cholecystitis.

An abdominal ultrasound revealed an approximately 8 mm gallstone with a moderate amount of sludge. There was subtle, generalized thickening of the gallbladder wall, but no pericholecystic fluid collection. The common bile duct (CBD) diameter was normal (Figure 1).

The patient was admitted with a diagnosis of acute calculous cholecystitis. On the third day of admission, he underwent a laparoscopic cholecystectomy. A drain was placed postoperatively, and empirical antibiotics (cefuroxime 1.5 g every eight hours and metronidazole 500 mg every eight hours) were initiated. Intraoperatively, the gallbladder was found to be distended and tense with omental adhesions over the liver surface. The cystic duct and CBD appeared dilated, with no contrast filling beyond the cystic duct.

**Figure 2:**
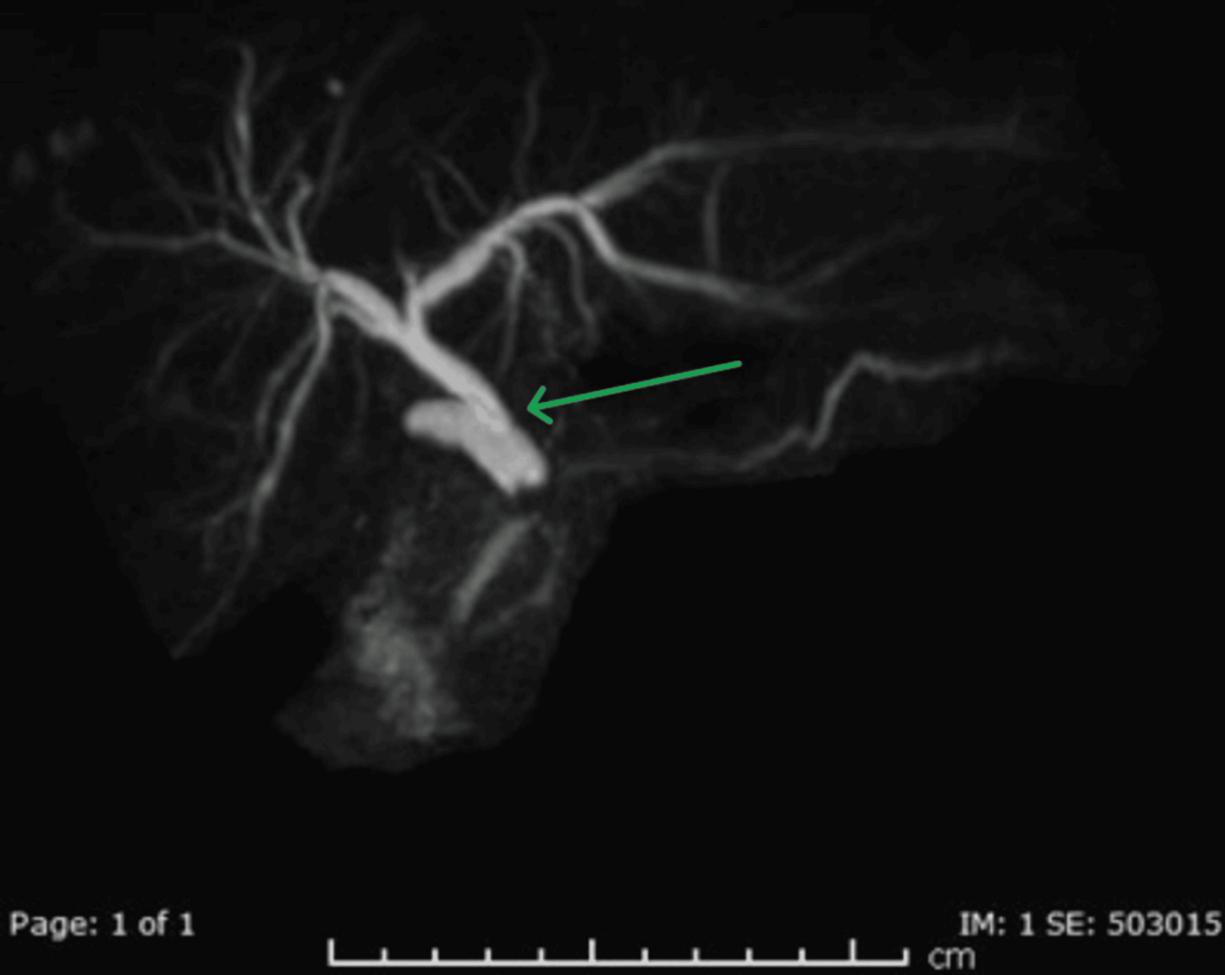
MRCP demonstrating cystic duct stump compression Magnetic resonance cholangiopancreatography (MRCP) shows a dilated long cystic duct stump with an impacted stone causing abrupt narrowing of the distal common bile duct (CBD) (green arrow). Associated dilatation of the proximal intrahepatic biliary ducts is also demonstrated.

**Figure 3:**
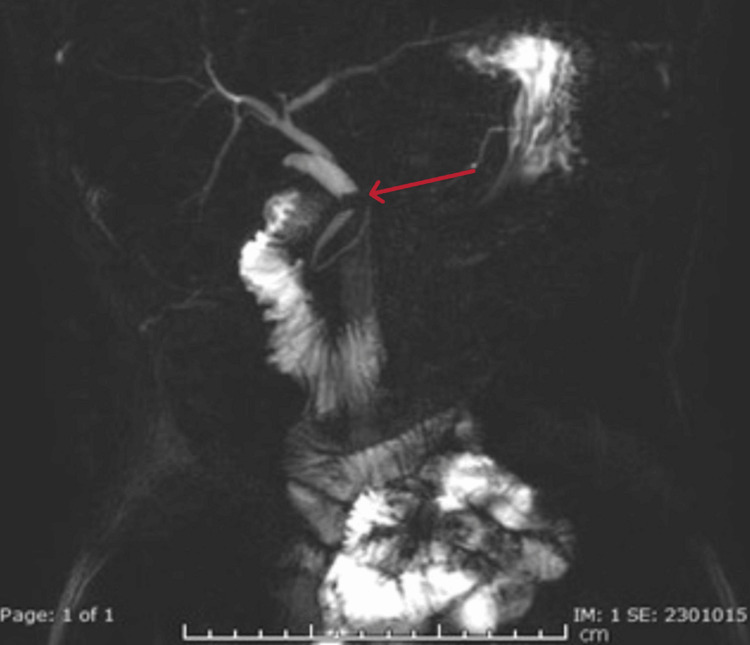
MRCP demonstrating impacted cystic duct stone Magnetic resonance cholangiopancreatography (MRCP) reveals an impacted cystic duct stone (red arrow) causing luminal narrowing of the distal common bile duct (CBD). Associated dilatation of the proximal CBD and intrahepatic biliary ducts is evident.

Following consultation with the gastroenterology team, an MRCP was performed on day seven. It showed no gallbladder (consistent with prior removal) but revealed a relatively long and dilated cystic duct stump (10.2 mm) with an impacted 5.5 mm stone near its junction with the CBD. The dilated cystic duct stump was compressing the CBD, which appeared mildly dilated proximally, along with dilated intrahepatic ducts, findings consistent with MS (Figures 2-3).

**Figure 4:**
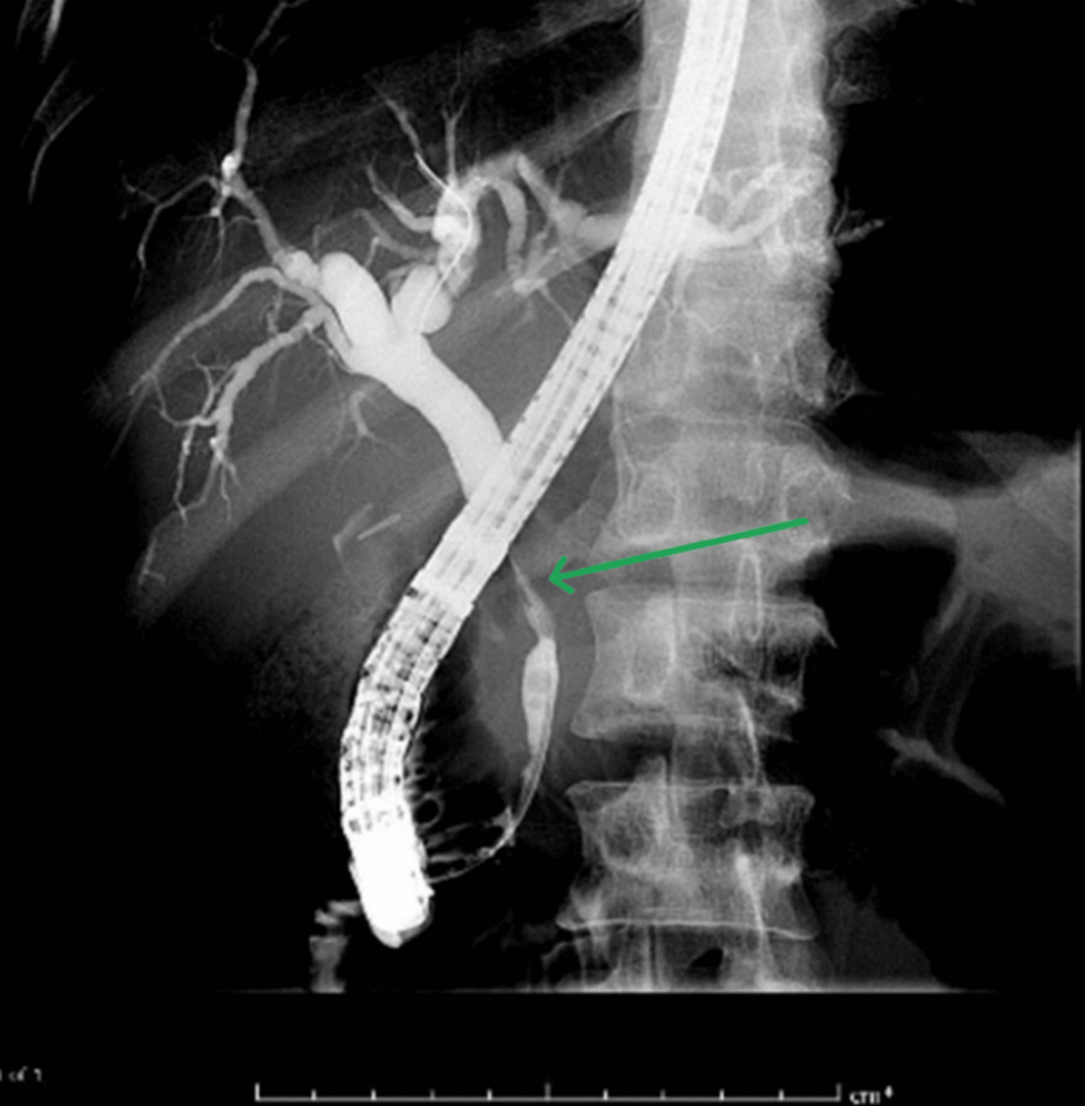
ERCP demonstrating biliary narrowing Endoscopic retrograde cholangiopancreatography (ERCP) shows a narrowing of the mid common bile duct (CBD) (green arrow) with associated obstruction and upstream dilatation of the hepatic biliary ducts.

**Figure 5:**
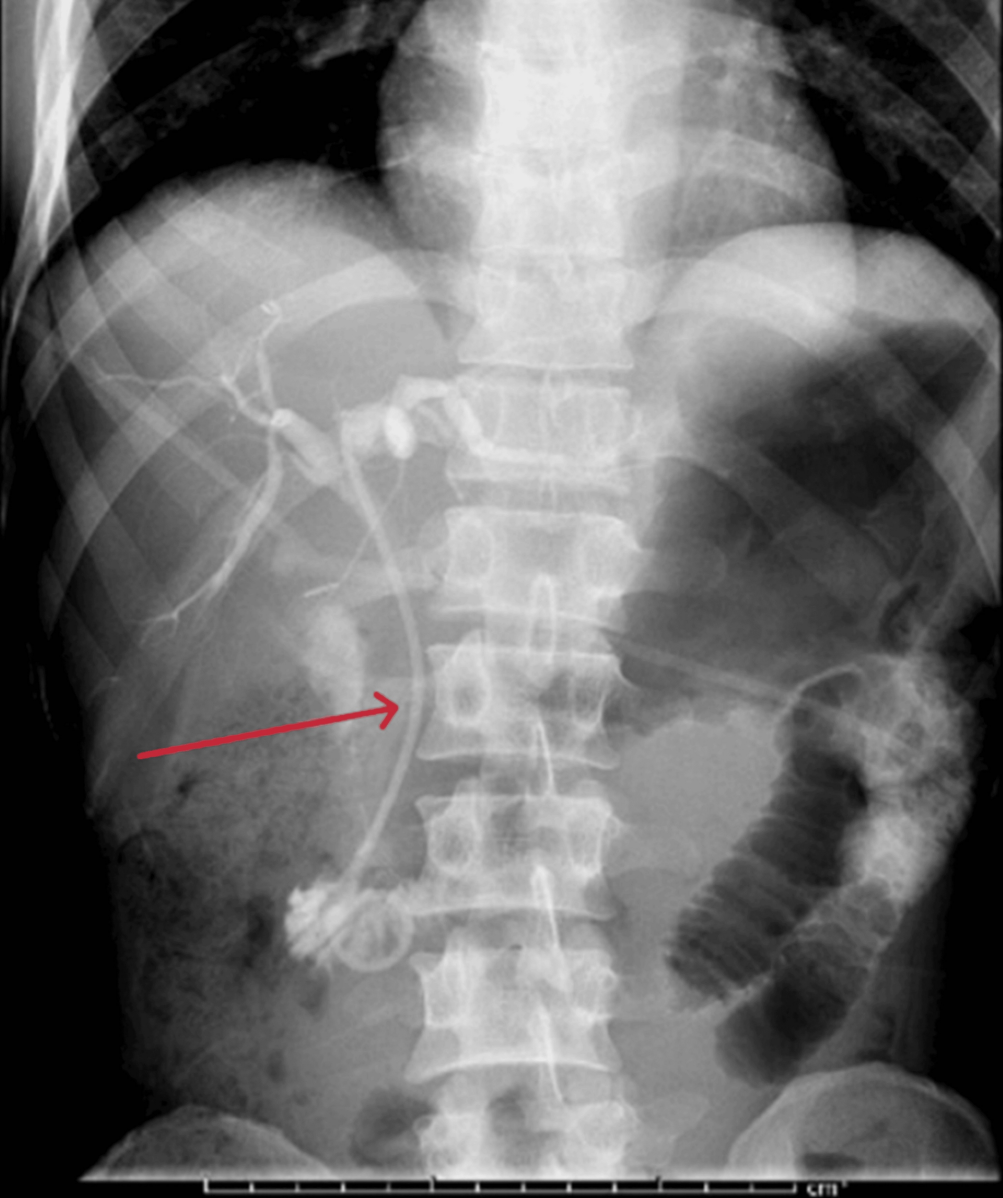
ERCP demonstrating biliary stent placement Endoscopic retrograde cholangiopancreatography (ERCP) shows the inserted plastic stent (red arrow) within the common bile duct, resulting in a significant reduction of the obstructive appearance of the hepatic biliary ducts.

After further discussion with gastroenterology, an ERCP was performed four days later, demonstrating a narrowing and filling defect in the proximal CBD just below the cystic duct insertion, with upstream CBD dilation and contrast hold-up, confirming MS (Figure 4). A sphincterotomy was performed, and a CBD stent was placed (Figure 5).

The patient was discharged in stable condition two days later. On follow-up visits at two, three, and five weeks post discharge, he remained clinically stable, with no new complaints. Stent removal was planned as the next step.

## Discussion

MS is a rare complication of gallstone disease that poses a significant diagnostic challenge, often mimicking conditions such as gallbladder cancer or acute cholecystitis. In this case, the patient had post-laparoscopic cholecystectomy symptoms and deranged LFT. Subsequent imaging with MRCP and ERCP confirmed an impacted stone at the distal part of the long cystic duct stump compressing the CBD, resulting in proximal biliary dilation. Timely intervention with ERCP, sphincterotomy, and CBD stenting effectively prevented complications such as cholangitis. This case highlights the importance of early and accurate recognition of MS, even when initial imaging appears inconclusive, to minimize morbidity and optimize outcomes through multidisciplinary care.

MS typically occurs when a gallstone becomes impacted in the cystic duct or infundibulum (Hartmann’s pouch) of the gallbladder, leading to chronic inflammation and external compression of the CBD or common hepatic duct [2]. This obstruction can result from a single large stone or multiple smaller stones, causing impaired bile flow and symptoms that overlap with acute cholecystitis, choledocholithiasis, or ascending cholangitis. Patients may present with jaundice, fever, and right upper quadrant pain, but these findings are not always present [3]. Preoperative diagnosis is frequently missed, particularly during laparoscopic cholecystectomy, increasing the risk of inadvertent biliary injury [3].

MS is reported in approximately 0.05%-4% of patients undergoing surgery for gallstones [1], with a higher prevalence in women, reflecting the overall gender distribution of cholelithiasis. Notably, MS is associated with an elevated risk of gallbladder cancer. Studies indicate that between 5% and 28% of patients with MS undergoing cholecystectomy may also harbor gallbladder malignancy [4]. The prevailing theory attributes this link to chronic inflammation and persistent biliary stasis. For instance, in a series of 4,800 cholecystectomy cases, MS was identified in 133 patients, of whom 5% were found to have gallbladder cancer, most detected intraoperatively or on postoperative pathology [5].

The classification of MS has evolved over time. Initially, McSherry proposed two types: type I, characterized by external compression of the CHD by an impacted stone, and type II, where the stone erodes into the CHD, forming a cholecystobiliary fistula. The Csendes classification later refined this into a more detailed system, subdividing cases based on the extent of bile duct involvement: type I involves only external compression; type II affects less than one-third of the CHD circumference; type III involves one-third to two-thirds; type IV represents complete circumferential destruction; and type V includes cases complicated by a cholecystoenteric fistula [6-9] (Table 2).

**Table 2:** Classification of Mirizzi syndrome with descriptions and reported incidence rates Description: Refs [6,7]; Incidence (%): Refs [8,9]

Classification of Mirizzi syndrome	Description	Incidence (%)
Type I	External compression of the common hepatic or bile duct by a gallstone impacted in the gallbladder neck or cystic duct.	10.5-78
Type II	A cholecystobiliary fistula forms where a gallstone erodes into and involves less than one-third of the common bile duct’s circumference	15-41
Type III	A cholecystobiliary fistula affects between one-third and two-thirds of the common bile duct.	3-44
Type IV	A cholecystobiliary fistula that completely involves and destroys the entire circumference of the common bile duct wall	1-4
Type V	Any type plus a cholecystoenteric fistula	29
Type Va	Without gallstone ileus	-
Type Vb	With gallstone ileus	-

Clinical presentation is highly variable. Although right upper quadrant pain, jaundice, and fever are classic features, they occur together in only about 44%-71% of cases [10]. Pain is the most common symptom, followed by jaundice, while cholangitis is less frequent. Many patients have a history of gallstones, and MS may present either acutely or with chronic symptoms. The average age at diagnosis ranges from 53 to 70 years, with a notable female predominance [11]. Other complaints may include nausea, vomiting, dark urine, and anorexia, and up to one-third of patients present with acute cholecystitis. A positive Murphy’s sign is found in roughly half of cases, while rare presentations such as gallstone ileus or a palpable mass have been documented [12]. Laboratory tests often reveal elevated alkaline phosphatase and bilirubin levels, although no laboratory findings are specific to MS [13].

Management primarily involves surgery to remove the inflamed gallbladder and any impacted stones. When MS is identified preoperatively, ERCP serves both diagnostic and therapeutic purposes. It allows for biliary decompression through stenting, especially in patients presenting with obstructive jaundice or cholangitis, and may help clear CBD stones, potentially avoiding CBD exploration [14]. If MS is discovered incidentally during cholecystectomy, intraoperative cholangiography is crucial for confirming the diagnosis and delineating biliary anatomy. In patients who are poor surgical candidates, ERCP with stenting may offer a definitive treatment option. Ultimately, the surgical approach depends on the type of cholecystobiliary fistula and the extent of duct involvement [15].

An important consideration is the overlap between MS and gallbladder cancer. Although serum CA 19-9 levels can aid in differentiation, they are not definitive, as elevations have been reported in benign MS cases [16]. The strong association between MS and gallbladder cancer, with prevalence estimates ranging from 5% to 28% [4], underscores the need for vigilance. This relationship is likely driven by chronic biliary stasis and recurrent inflammation. In large surgical series, gallbladder cancer was often only diagnosed intraoperatively or on postoperative pathology [5], highlighting the challenge of distinguishing these conditions before surgery.

## Conclusions

MS remains a diagnostic challenge due to its nonspecific presentation and similarity to other biliary conditions. Early recognition through a combination of clinical suspicion and advanced imaging is essential to prevent complications such as bile duct injury and cholangitis. This case highlights the importance of the risks of a long cystic duct stump (which should be less than 10 mm and that is enough to safely apply clips without compromising the CBD); the risks include post-cholecystectomy syndrome, stump stone formation, and MS type 1. The case also highlights the use of a multidisciplinary approach involving surgery and gastroenterology to achieve optimal management. Awareness of MS, especially in patients with gallstones and atypical biliary symptoms, can improve outcomes and reduce morbidity.
